# Effect of prehospital transportation on 24-h fluid volume, a *post hoc* analysis of a multicenter, prospective, observational study on fluid volumes in patients with suspected infection

**DOI:** 10.3389/fmed.2022.1052071

**Published:** 2022-12-21

**Authors:** Marie Egebjerg Jensen, Jens Aage Kølsen-Petersen, Hans Kirkegaard, Marie Kristine Jessen

**Affiliations:** ^1^Research Center for Emergency Medicine, Department of Clinical Medicine, Aarhus University and Aarhus University Hospital, Aarhus, Denmark; ^2^Department of Anesthesiology and Intensive Care, Aarhus University Hospital, Aarhus, Denmark; ^3^Prehospital Emergency Medical Services, Aarhus, Denmark; ^4^Department of Emergency Medicine, Aarhus University Hospital, Aarhus, Denmark

**Keywords:** Emergency Medical Services (EMS), emergency department, fluid therapy, sepsis, infection

## Abstract

**Introduction:**

Infections, including sepsis, are leading causes of death and fluid administration is part of the treatment. The optimal fluid therapy remains controversial. If the patient is transported by Emergency Medical Services (EMS), fluids can be initiated during transportation, which may result in increased overall fluid administration and fluid overload, which may be harmful. The aim of the study was to investigate the effect of EMS transportation on 24-h fluid administration in patients with suspected infection.

**Methods:**

This is a *post hoc* study of a prospective, multicenter, observational study, conducted in three Danish Emergency Departments (EDs), 20 January–2 March 2020, aiming at describing fluid administration in patients with suspected infection. Patients were stratified into the groups: simple infection or sepsis, in accordance with SEPSIS-3-guidelines. The primary outcome of the current study was 24-h total fluid volume (oral and intravenous) stratified by transportation mode to the EDs.

**Main results:**

Total 24-h fluids were registered for 734 patients. Patients with simple infection or sepsis arriving by EMS (*n* = 388, 54%) received mean 3,774 ml (standard deviation [SD]: 1900) and non-EMS received 3,627 ml (SD: 1568); mean difference (MD) was 303 ml [95% CI: 32; 573] adjusted for age, site, and total SOFA-score. Patients brought in by EMS received more intravenous fluids (MD: 621 ml [95% CI: 378; 864]) and less oral fluids (MD: -474 ml [95% CI: −616; −333]) than non-EMS patients.

**Conclusion:**

Patients transported by EMS received more intravenous fluids and less oral fluids but overall, more fluid in total in the first 24-h than non-EMS after adjusting for age, site and SOFA-score.

## Introduction

Suspected infection and sepsis is a global health problem. Annually, 30–50 million people suffer from sepsis with 11 million reported sepsis-related deaths, representing 20% of global mortality–not even including patients with suspected infection without sepsis (1). Up to 25% of patients admitted with suspected infection or sepsis progress into septic shock (2), which carries an even higher mortality (3). To reduce morbidity and mortality from infections and sepsis, early diagnosis and initiation of treatment is recommended including fluid administration and antibiotics, but fluid volumes remain controversial (4, 5).

Surviving Sepsis Campaign recommends giving 30 ml/kg intravenous fluids within the first 3 h of resuscitation (weak recommendation, low quality of evidence) to patients with sepsis-associated hypotension and septic shock, but the guidelines do not give any recommendations to neither patients with simple infection nor sepsis without hypotension or shock, although sepsis without hypotension or shock is approximately 60 times more common than septic shock (5, 6).

The majority of sepsis patients are admitted through the emergency department (ED) and 35–60% of these patients are brought in by ambulance by Emergency Medical Services (EMS) (7–9). Sepsis patients arriving by ambulance are seen faster by ED physicians, sepsis is recognized faster, and the antibiotic treatment is initiated earlier than for patients not arriving by ambulance (9–12). Whether this is the same for patients with simple infection is unknown. During transportation, patients often receive intravenous (IV) fluid in the ambulance before reaching the hospital. This early treatment may benefit patients. On the other hand, studies have indicated that an overall restrictive fluid administration could improve outcomes in patients with sepsis associated hypotension or shock (13–15). If early initiation of fluids during EMS transportation results in accelerated, increased volumes of fluid and thereby a risk of fluid overload, this could possibly harm the patient. With no existing guidelines for fluid administration in neither patients with simple infection nor patients with sepsis without hypotension or shock and a heterogenous population of infected patients it is difficult for prehospital and ED personnel to uniform treatment. It has, though, never been examined, if being brought in by EMS influences the total fluid volume administered within the first 24 h of hospitalization.

We aimed to examine the differences in fluid administration during the first 24 h in-hospital in patients with suspected infection brought in by EMS or arriving on their own and hypothesized that patients brought in by EMS received more fluids than non-EMS patients.

## Materials and methods

### Study design

This is a *post hoc* study of a prospective, observational study conducted at three EDs and hospitals in Central Denmark Region: Aarhus University Hospital, Regional Hospital Herning and Region Hospital Randers from 20 January 2020, through 2 March 2020 (16). The original study aimed at describing current 24-h fluid administration to all ED patients admitted with suspected infection of any severity. This manuscript has been prepared in accordance with the Strengthening the Reporting of Observational Studies in Epidemiology (STROBE) statement (17). The completed checklist is presented in the supplement.

### The original study

The study was a prospective, multicenter, observational study. All ED patients at the three sites were screened for eligibility during the study period. We consecutively included all patients who fulfilled all of the following inclusion criteria: age ≥ 18 years, admitted through the ED with suspected infection defined as drawing of a blood culture and/or administration of IV antibiotics within 6 h of arrival (16). We only included Danish citizens with a personal identification-number to be able to track them in the electronic patient record. Only the first presentation with all inclusion criteria fulfilled within the study period was included. We excluded (1) patients who were admitted after severe trauma, (2) patients with serious bleeding defined by the use of more than two units of red blood cells or the need for an invasive intervention for bleeding, and (3) patients, who only received prophylactic antibiotics (e.g., patients scheduled for surgery) who did not have a blood culture drawn.

#### Fluid registration

All included patients had oral and IV fluids registered on a paper case report form (CRF, see original article) for the first 24 h of their hospital stay including fluids administered in the prehospital setting. All intravenous fluids ≥ 50 ml were registered. Intravenous fluids included crystalloids, glucose, albumin, parenteral nutrition, and blood products. Oral fluids were registered by the treating nurses and/or by the patient if deemed fully conscious and cooperative. Tube feeding was registered as part of oral fluids. For all fluids–oral and intravenous–the administration start time was noted. The CRF followed the patient for 24 h or until discharge within 24 h. In the analyses, we only included patients who were admitted for the entire 24-h time-period with fluid administration registered.

#### Illness severity and other variables

Patients were divided into groups of illness severity within 6 h of ED arrival: simple infection (SOFA-score < 2), sepsis (increase in SOFA-score ≥ 2 from baseline), and septic shock [mean arterial pressure (MAP) of 65 mmHg or greater and serum lactate level > 2 mmol/L (>18 mg/dL)] based on the Sepsis-3 guidelines using SOFA-score (Sequential Organ Failure Assessment-score) (18). Descriptive data on vital signs, organ dysfunction, receipt of and timing of intravenous antibiotics, comorbidities, mortality, ED length of stay, and in-hospital length of stay were automatically retrieved from the electronic patient record at each hospital.

### Setting

In the three EDs, patient contacts vary between 15,000 and 63,000 per year with a mixed rural-urban population providing 24-h emergency care to all acute patients except those transferred directly to catheterization laboratory (ST-elevation myocardial infarction patients), stroke units (thrombolysis candidates), and women in labor. Healthcare, including prehospital care, in Denmark is tax-supported, subsidizing equal access to hospital treatment regardless of income.

### The current *post hoc* study

This *post hoc* study investigated the effect of prehospital transportation on fluid treatment in patients with suspected infection. All in- and exclusion criteria were the same as for the original study. The study was approved by the Danish Patient Safety Authority (case no.: 31-1521-188 and case no.: 1-45-70-69-20). Approval from an ethics committee was not required according to Danish law.

### Prehospital transportation

For every included patient, patient transportations were for this *post hoc* study divided into either “EMS transportation” if the patient was brought in by ambulance or “non-EMS transportation” for patients arriving by themselves, by taxi or a non-urgent lying or sitting transportation.

Emergency Medical Services transportation was identified, and data abstracted from the electronic Prehospital Patient Journal (PPJ) containing individual-level information about the urgency, dispatch criteria, on-scene timestamps for each EMS event using the unique patient identification number (the Civil Personal Registration number). Information about prehospital complaints or symptoms were obtained from the EMS notes in PPJ, and the different symptoms were categorized into nine groups defined *a priori*. Prehospital fluid administration was included in IV fluids 0–6 h.

### The EMS system

The Danish EMS is a two-tiered ambulance system consisting of ambulances as the basic-level response (staffed by paramedics and trained emergency medical technicians) and an advanced level with rapid response vehicles (staffed by anesthesiologists and paramedics) or helicopters (staffed with anesthesiologist, paramedics, and a pilot). Denmark is served by regionally organized systems of EMS dispatchers. All medical calls are directed to one of five public emergency medical coordination centers staffed by healthcare professionals determining the urgency of the transportation (19). The acute EMS transportation is categorized by severity; A with the highest urgency (life threatening), B as urgent but not immediately life threatening and C as non-critical.

### Outcomes

The primary outcome was total fluid volume (all oral and intravenous combined) within the first 24 h of hospital admission including fluid administered during prehospital transportation. Secondary outcomes were total fluids 0-6 h, and intravenous as wells as oral fluids, respectively, in 0–6 and 0–24 h.

#### Study size and statistics

For the original study, we did not calculate a formal sample size *a priori*, but based on previously unpublished data from Aarhus University Hospital, we anticipated that approximately 1,600 patients with suspected infection would present at the three sites within the study period. This anticipated sample size was deemed adequate for the descriptive goal of the study. For this *post hoc* study, we anticipated that approximately 35–60% of all included patients were transported by EMS (7, 8), still deemed adequate for the descriptive purpose.

For all analyses on fluid volumes and differences in these, we included all patients with simple infection or sepsis (*n* = 726) from the original study in one combined group but did not investigate the septic shock patients (*n* = 8). This was chosen since the original study showed no statistically significant difference in fluid volumes between patients with simple infection or sepsis, and the septic shock groups was very small and all patients were transported by EMS (16). However, baseline characteristics are reported for the analyzed groups and for all illness severity sub-groups to ensure transparency of patient characteristics across the severity groups. Since all septic shock patients were transported by EMS, we did not investigate differences in fluid volumes in this small patient group.

Categorical data are reported as counts and proportions (%) and continuous data as means (standard deviations [SD]) or medians (1st and 3rd quartile, interquartile range [IQR]), as appropriate. To assess the association between EMS transportation vs. non-EMS transportation and total fluid volume, linear regression models were used with the amount of fluid within the first 24 h as the primary outcome; these analyses were subsequently adjusted for site, age and illness severity and reported with 95% confidence intervals. Distributions were assessed for normality using visual inspection of histograms. Age and illness severity were chosen since they were found to be confounders in the primary study (16). This was also performed for total fluids 0–6 h, and both 0–6 and 24-h oral and intravenous fluids. There was no missing data on neither primary or secondary outcomes nor any of the adjusting variables for any patients. Data were analyzed using Stata version 16 (StataCorp LP, College Station, TX, USA).

## Results

### Characteristics of patients

**FIGURE 1:**
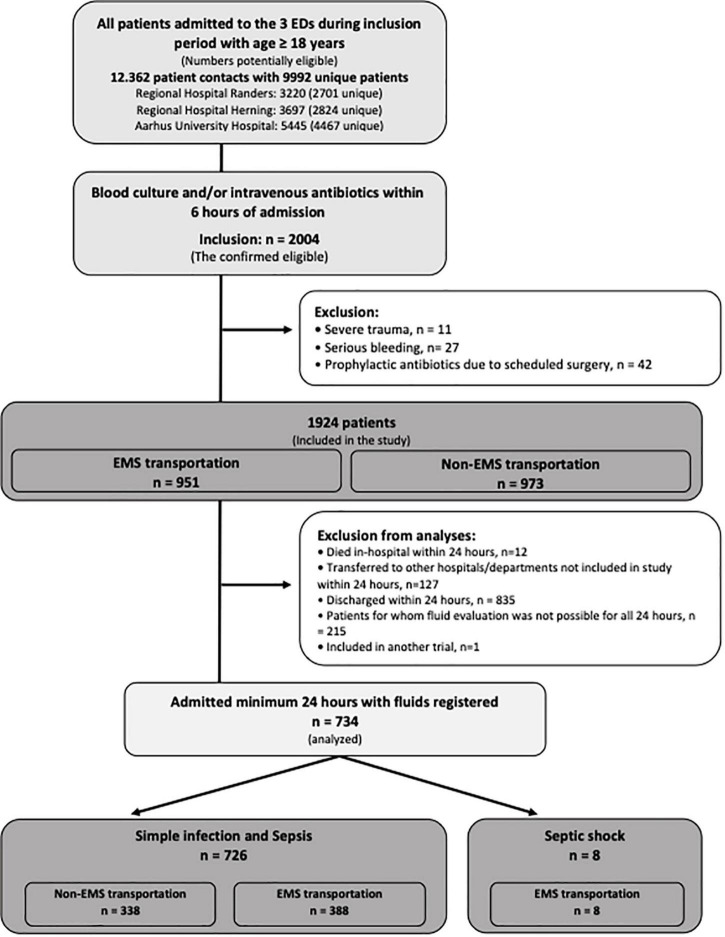
Flow chart of this figure shows patient flow and transportation status.

Total 24-h fluids were registered in 734 patients: 387 with simple infection, 339 with sepsis, and 8 with septic shock. Looking at patients with simple infection or sepsis combined, 388 (53%) were brought in by EMS. For complete patient flow, see Figure 1. The baseline characteristics are shown in Tables 1, 2. Patients brought in by EMS were in general older than patients arriving on their own, and the infectious source was more often respiratory and less often abdominal. Patients brought in by EMS tended to have higher in-hospital mortality even though the patients dying within the first 24 h were excluded from the analyses. The majority of EMS patients were transported as urgency B, but 7/8 (88%) septic shock patients were urgency A. Characteristics of their prehospital transportation including urgency of transportation if brought in by EMS are shown in Table 3.

**TABLE 1:** Baseline characteristics of patients admitted minimum 24 h with fluids registered.

	Simple infection (*n* = 387)		Sepsis (*n* = 339)		Septic shock (*n* = 8)
Variable	Non-EMS transportation (*n* = 232)	EMS transportation (*n* = 155)	Non-EMS transportation (*n* = 106)	EMS transportation (*n* = 233)	EMS transportation (*n* = 8)
Age, years	66 [46;76]	78 [67;86]	73 [57;80]	78 [69;83]	75 [55;76]
Male sex, *n* (%)	103 (44%)	83 (55%)	57 (53%)	139 (59%)	3 (38%)
Weight, kg	75 [63;88]	74 [62;86]	78 [62;90]	75 [61;87]	71 [60;87]
BMI, kg/m^2^	25 [22;29]	25 [22;29]	26 [23;30]	25 [22;28]	25 [24;28]
DNR/DNI at admission	15 (6%)	32 (20%)	20 (19%)	85 (25%)	2 (19%)
Known heart failure^a^	7 (3%)	9 (6%)	21 (9%)	7 (7%)	1 (13%)
Known renal disease^b^	27 (12%)	18 (12%)	11 (10%)	37 (16%)	0 (0%)
**Vital signs, worst within 6 h**					
Respiratory rate, min^–1^	18 [16;22]	20 [18;25]	22 [18;28]	22 [20;28]	27 [19;30]
Saturation, %	96 [94;98]	95 [93;97]	93 [90;96]	92 [88;95]	95 [89;99]
Heart rate, min^–1^	93 [84;106]	93 [80;107]	98 [85;111]	98 [85;117]	101 [71;117]
Systolic BP, mmHg	132 [117;144]	134 [111;149]	121 [107;137]	115 [99;134]	103 [87;114]
MAP, mmHg	92 [79;102]	92 [84;102]	89 [75;98]	83 [71;96]	74 [64;80]
Temperature, °C	38.1 [37.6;38.8]	38 [37.4;38.6]	38.2 [37.6;38.2]	38.1 [37.4;38.8]	36.2 [35;37.1]
GCS	15 [15;15]	15 [15;15]	15 [15;15]	15 [14;15]	8 [5;11]
**Blood tests**					
Creatinine, μmol/l	69 [58;95]	73 [58;91]	79 [62;112]	88 [66;129]	101 [66;132]
Platelets, ×10^9^/l	270 [211;342]	251 [200;311]	222 [173;274]	214 [169;294]	281 [228;357]
Billirubin, μmol/l	10 [7;13]	8 [6;14]	13 [8; 24]	12 [7;20]	8 [4;12]
Leukocytes, ×10^9^/l	11 [8;14]	12 [9;15]	12 [9;16]	11 [9.4;16]	11 [7;17]
C-reactive protein, mg/l	112 [43;197]	62 [20;135]	95 [43;232]	60 [20;142]	17 [7;57]
Lactate, mmol/l	1.4 [0.8;1.7]	1.6 [1;2.4]	1.5 [1.1;2.2]	1.5 [1;2.6]	8.5 [3.8;10.2]
Hemoglobin, mmol/l	7.9 [7.1;8.6]	7.8 [7;8.5]	7.9 [6.9;8.8]	7.9 [6.8;8.7]	8.5 [7.7;9]
Glucose, mmol/l	6.4 [5.7;7.4]	6.8 [6;8.2]	7.7 [6.4;9.9]	7.5 [6.5;9.6]	11.9 [6.9;15.7]
Blood culture drawn, *n* (%)^c^	229 (99%)	143 (92%)	103 (97%)	218 (94%)	8 (100%)
Time to blood culture, h	0.6 [0.4;0.8]	0.4 [0.3;0.7]	0.5 [0.3;0.8]	0.4 [0.2;0.7]	0 [0.0;0.4]
IV AB, *n* (%)**	87 (38%)	68 (44%)	48 (45%)	116 (50%)	5 (63%)
Time to IV AB, h	2.3 [1.3;4.0]	1.8 [0.7;4,6]	1.8 [0.7;3.3]	1.4 [0.5;3.0]	0.3 [0.3;0.5]
Oral AB administered, *n* (%)	23 (10%)	11 (7%)	5 (5%)	16 (7%)	0 (0%)

All data are presented as medians with IQR if not otherwise stated. ^a^A known ejection fraction < 40% before admission. ^b^Renal disease of any kind affecting renal function. ^c^Drawn or administered within 6 h of arrival. BMI, body mass index; DNR, do-not-resuscitate; DNI, do-not-intubate-order; MAP, mean arterial blood pressure; BP, blood pressure; GCS, Glasgow Coma Scale (Range 3–15); IV, intravenous; AB, antibiotics. **Administered within 6 hours of admission.

**TABLE 2:** Baseline characteristics of patients admitted minimum 24 h with fluids registered.

	Simple infection (*n* = 387)		Sepsis (*n* = 339)		Septic shock (*n* = 8)
Variable	Non-EMS transportation (*n* = 232)	EMS transportation (*n* = 155)	Non-EMS transportation (*n* = 106)	EMS transportation (*n* = 233)	EMS transportation (*n* = 8)
qSOFA	0 [0;1]	1 [0;1]	1 [0;1]	1 [1;2]	2 [2;3]
**SOFA-score^a^**					
Total SOFA-score	0 [0;1]	0 [0;1]	2 [2;3]	3 [2;4]	10 [9;12]
Respiration	0 [0;0]	0 [0;0]	2 [0;2]	2 [1;2]	3 [2;3]
Coagulation	0 [0;0]	0 [0;0]	0 [0;1]	0 [0;1]	0 [0;0]
Liver	0 [0;0]	0 [0;0]	0 [0;0]	0 [0;0]	0 [0;0]
Cardiovascular	0 [0;0]	0 [0;0]	0 [0;0]	0 [0;0]	4 [3;4]
Central nervous system	0 [0;0]	0 [0;0]	0 [0;0]	0 [0;1]	3 [2;3]
Renal	0 [0;0]	0 [0;0]	0 [0;0]	0 [0;1]	1 [0;1]
**Infectious source, *n* (%)**					
Respiratory	50 (22%)	50 (32%)	40 (37%)	101 (43%)	3 (38%)
Urinary	46 (20%)	35 (23%)	12 (11%)	42 (18%)	1 (13%)
Skin/soft tissue	38 (16%)	10 (6%)	7 (7%)	7 (3%)	0 (0%)
Abdominal	42 (18%)	20 (13%)	22 (21%)	18 (8%)	1 (13%)
Bacteremia–no other source	1 (0.1%)	0 (0%)	1 (0.9%)	0 (0%)	0 (0%)
Viral	9 (4%)	7 (5%)	5 (5%)	8 (3%)	1 (13%)
Other^b^	13 (6%)	5 (3%)	3 (3%)	8 (3%)	1 (13%)
Unknown	12 (5%)	15 (10%)	8 (8%)	24 (10%)	0 (0%)
No suspected infection^c^	21 (9%)	13 (8%)	8 (8%)	25 (11%)	1 (13%)
ED length of stay, h	12 [7;25]	16 [7;26]	15 [7;23]	13 [6;28]	1 [0.6;1.6]
In-hospital length of stay, h	77 [48;130]	110 [63;170]	118 [71;166]	124 [71;196]	212 [154;575]
In-hospital mortality, *n* (%)	3 (1%)	6 (4%)	6 (6%)	15 (6%)	0 (0%)
90-day mortality, *n* (%)	16 (7%)	49 (21%)	20 (19%)	49 (21%)	0 (0%)

All data are presented as medians with IQR if not otherwise stated. ^a^SOFA-score is defined per Sepsis-3-guidelines. ^b^Other infections included neurological, cardiovascular, gynecological, ear-nose-throat, etc. ^c^Suspected or documented infection not mentioned in charts, but patient fulfilled the inclusion criteria in the study for suspected infection (blood culture and/or intravenous antibiotics). qSOFA, quick Sequential Organ Failure Assessment; SOFA, Sequential Organ Failure Assessment; ED, emergency department.

**TABLE 3:** Prehospital characteristics of patients admitted minimum 24 h with fluids registered.

	Simple infection (*n* = 387)	Sepsis (*n* = 339)	Septic shock (*n* = 8)
Prehospital ambulance transportation, *n* (%)	155 (40%)	233 (69%)	8 (100%)
**Type of ambulance transportation, *n* (%)**			
A	42 (27%)	92 (39%)	7 (88%)
B	68 (44%)	100 (43%)	1 (13%)
C	45 (29%)	41 (18%)	0 (0%)
Ambulance transportation time, min^a^	35 [29;46]	38 [29;50]	46 [37;58]
**Main prehospital symptom, *n* (%)**			
Dyspnea	34 (22%)	87 (38%)	0 (0%)
Abdominal or vomiting	32 (21%)	24 (10%)	2 (25%)
Headache, unconsciousness, or seizures	14 (9%)	40 (17%)	4 (50%)
Unspecific uncomfort or dehydration	22 (14%)	27 (12%)	1 (13%)
Fever	22 (14%)	26 (11%)	0 (0%)
Heart symptoms	13 (9%)	8 (3%)	1 (13%)
Urinary tract symptoms	10 (6%)	16 (7%)	0 (0%)
Trauma	3 (2%)	1 (0.5%)	0 (0%)
Other^b^	5 (3%)	4 (2%)	0 (0%)

All data are presented as medians with interquartile range [IQR] if not otherwise stated. ^a^Time from first EMS patient contact to arrival to the ED. ^b^Other: Includes: Pain in extremities, backpain, fall at home, anemia, etc.

### The effect of prehospital transportation on fluid administration

Patients with simple infection or sepsis brought in by EMS (*n* = 726) received 3,774 ml (Mean, SD: 1900) and non-EMS patients received 3,627 ml (SD: 1568) of total oral and intravenous fluids in 24 h. Unadjusted, there were no significant differences between these two groups {Mean difference (MD): 147 ml [95% CI: −109; 403]}, but after adjusting for age, site and total SOFA-score (illness severity), patients brought in by EMS received 303 ml [95% CI: 32; 573] more total fluid than non-EMS within 24 h (Table 4).

**TABLE 4:** Fluid administration in the first 24 h stratified by Emergency Medical Services (EMS) or non-EMS transportation for patients with simple infection or sepsis combined.

	EMS transportation (*n* = 388)	Non-EMS transportation (*n* = 338)	MD between EMS and non-EMS *trans* (Unadjusted)	*P*-values	MD between EMS and non-EMS *trans* (Adjusted^a^)	*P*-values
**Total fluid volume in ml**						
T0–T24 h, mean (SD)	3,774 (1,900)	3,627 (1,568)	147 [-109;403]	0.261	303 [32;573]	0.028
T0–T6 h, mean (SD)	1,698 (1,060)	1,526 (825)	171 [32;311]	0.016	228 [79;376]	0.003
**Total intravenous fluid volume in ml**						
T0–T24 h, mean (SD)	2,396 (1,861)	1,775 (1404)	621 [378;864]	<0.001	477 [221;733]	<0.001
T0–T6 h, mean (SD)	1,332 (1,025)	916 (749)	386 [253;518]	<0.001	296 [156;437]	<0.001
**Total oral fluid volume in ml**						
T0–T24 h, mean (SD)	1,378 (840)	1,852 (1,091)	-474 [-616;-333]	<0.001	-175 [-321;-28]	0.020
T0–T6 h, mean (SD)	396 (429)	610 (533)	-214 [-284;-144]	<0.001	-69 [-142;5]	0.067

Results are given in means (±standard deviation [SD]) or mean differences (MD) [95%CI]. MD, mean difference. ^a^MD [95%CI] adjusted for site, age, and total SOFA-score.

Looking at only intravenous fluid in 24 h, patients brought in by EMS received significantly more fluids than non-EMS patients (*n* = 726): EMS: 2396 ml (Mean, SD: 1861) and non-EMS: 1775 ml (SD: 1404), MD: 621 ml [95% CI: 378; 864]. Adjusted for site, age, and total SOFA-score the difference remained significant: MD: 477 ml [95% CI: 221; 733]. Also, for 6-h total and 6-h intravenous fluids, EMS-patients received more fluid than non-EMS patients (Table 4).

When looking at oral fluid, patients brought in by EMS received less oral fluid in 24 h than patients arriving on their own, MD: −474 ml [95% CI: −616; −333] and −175 ml [95% CI: −321; −28] after adjusting for site, age and total SOFA-score. For the first 6 h, MD in 6-h oral fluid volume was -214 ml [95% CI: −284; −144], but there was no difference, MD: −69 ml [95% CI: −142; 5] after adjustment (*n* = 726).

### The effect of prehospital transportation urgency on fluid administration

In patients brought in by ambulance with simple infection or sepsis, there was no difference in 24-h fluid administration between patients with transportation urgency A and B and C after adjusting for age, site, and illness severity (Supplementary Table 1) (*n* = 726). However, urgency B received more fluid than C when looking at 6-h intravenous fluid, MD: 303 ml [95% CI: 45; 562] and 6-h total fluid, MD: 277 ml [95% CI: 13; 542].

## Discussion

Patients with simple infection or sepsis brought into the emergency department by EMS received approximately 300 ml more fluid in 24 h than patients arriving on their own after adjusting for the known confounders age and illness severity. Patients brought in by EMS received almost 500 ml more intravenous fluids and approximately 200 ml less oral fluids than patients arriving on their own.

Patients transported by ambulance were older than patients arriving on their own, which has been found in several studies (9, 10). In accordance with the original study in this cohort, this *post hoc* secondary study found age to be a confounder of fluid volume with decreasing fluid volumes with increasing age (16). The age differences between patients in the non-EMS transportation group and the EMS transportation group (68 years [IQR:51;78] vs. 78 years [IQR:68;85], respectively) seem to impact and at least partly explain the differences in total fluids. Age is associated to increasing risk of heart failure which may lead physicians to reduce fluid volumes. However, a hypothesis generating Dutch multicenter, observational study suggested that older patients with suspected infection may need higher fluid volumes than younger patients to avoid in-hospital death, even when having a seemingly normal systolic blood pressure at admission (20). We did not adjust for comorbidities such as heart failure, known renal failure nor other comorbidities. We chose not to adjust for these, since they were not found to be significantly associated with fluid volumes in our original study, although it may reflect small numbers of patients with these comorbidities rather than no true effect. We acknowledge that these comorbidities can be potential confounders that might affect differences in fluid volumes found especially in our adjusted analyses.

Early initiation of fluid treatment can in patients brought in by EMS, possibly influence the patient outcomes in contradictory ways. Initiating the fluid treatment early is in general recommended to reduce mortality (5), giving EMS transported patients a possible benefit by initiating fluid therapy already before reaching the hospital (10). The effect of prehospital fluid treatment on mortality in patients with sepsis is, however, unknown; Seymour and colleagues found that the administration of any prehospital fluid was associated with reduced hospital mortality (odds ratio: 0.46; 95% CI: 0.23–0.88) compared to no prehospital fluid in sepsis patients (21). In line with this, an Australian observational study found each 1,000 ml increase in intravenous fluids associated with a risk reduction of in-hospital mortality in ED patients with suspected infection of any severity (22). In contrary, a more recent study from Lane and colleagues found that sepsis patients receiving any prehospital fluid had increased mortality (odds ratio, 1.3; 95% CI, 1.0–1.6) compared with sepsis patients not receiving any prehospital fluid. But in the same study they found that early administration of intravenous fluid was associated with a reduced mortality in patients with sepsis who had a low initial blood pressure (<100 mmHg) (23). Neither of the studies did, however, describe the in-hospital treatment nor report total 6- or 24-h fluids including in-hospital fluids.

Emergency Medical Services-personnel have varying knowledge of sepsis with a possibility of causing heterogeneous, individual treatments (24). Also the sepsis population is heterogenous with a variety of chief complaints when presenting to the prehospital EMS personnel (25). In a survey, ambulance personnel faced challenges deciding on fluid volumes and individual fluid needs and requested education, research and evidence-based guidelines (24). A study by Guerra and colleagues showed that by training EMS-personnel in recognizing severe sepsis, the mortality was reduced to 14% from 27% for patients treated by the trained EMS-personnel (26). Whether this education changed fluid strategies is, however, not reported. Overall, studies suggest that EMS transportation starts a trajectory with multiple interventions, for example, faster blood culture, earlier intravenous antibiotics and time to triage or physician (9–11, 27) and specifically reported in one of the studies in a reduction in time to IV fluids to 34 with EMS transportation from 68 min without EMS transportation. Likewise, our study suggests that this trajectory leads to initiation of fluids in patients with suspected infection.

Previous studies have found that ED patients brought in by EMS are sicker than those arriving on their own with higher severity scores and more organ failures after adjusting for demographics and co-morbidities (8, 10). Our study suggests higher total SOFA-score in the patients brought in by EMS with simple infection or sepsis, with the respiratory SOFA-score having the largest impact, although we recognize that SOFA-score is not the best way to describe illness severities in this rather old, comorbid population with high DNI/DNR-rates. The 24- and, also, 6-h fluid mean differences were statistically significant after adjusting for illness severity i.e., SOFA-score.

Patients brought in by EMS versus arriving on their own turned out to have different prehospital complaints or symptoms and documented infectious sites with respiratory focus and abdominal focus as the predominant, respectively. A Danish study looking at prehospital transportation of sepsis patients also found lower respiratory tract infections to be frequent in ambulance-transported patients with 67% vs. 59% for non-ambulance transportation, whereas abdominal infections were more frequent in non-ambulance transported patients (12% vs. 9.7%), in accordance with our findings (7).

The latest Sepsis Surviving Campaign guideline does, as described, not give recommendations for fluid administration of neither patients with simple infection nor sepsis patients without sepsis-associated hypotension or shock (5). In the septic shock population, the large-scale CLASSIC trial–a multicenter trial of 1,554 septic shock patients in the ICU randomized to restrictive fluids or standard care during their entire ICU-stay–recently found no difference in 90-day mortality, adding to the knowledge from a systematic review which also did not find differences in outcomes between restrictive and liberal fluids (15, 28). These findings leave clinicians with an opportunity to interpret the results according to own beliefs on fluids; either you can conclude that fluids are “unnecessary” and you can as well just treat the patients restrictively, or you can conclude that fluids also did not make it worse and fluids can be infused as you like or think the patient somehow would benefit from. Patients in the CLASSIC trial did, however, receive approximately 3,000 ml in the 24 h before randomization (28). This has in the critical care societies raised the question, whether the restrictive approach would be beneficial if initiated prior to administration of the rather high pre-enrollment fluid volumes. This questions the use of fluid for ED patients with less severe infections and therefore also raises the question if fluids should in general be administered prehospitally at all in this patient group since it seems to increase the total fluid volumes. Also, it could be that oral fluids should be emphasized over intravenous fluids in this patient group, if possible. Given that a large proportion of ED sepsis patients are brought in by EMS, it is important for the EDs to collaborate closely with the EMS to improve care also in future studies.

Due to the uniformly organized Danish public healthcare system, all patients included in the study were identified in the population-based registers for both ambulance and hospital medical records. Therefore, baseline characteristics and transportation on all patients meeting the inclusion criteria are presented and in general, data originates from a well-conducted, prospective, multicenter, observational study.

## Limitations

Fluid registration on the paper-CRFs was initiated as soon as possible after the patient fulfilled the inclusion criteria on arrival to the ED and at least within 6 h. An advantage of this setup was that prehospital personnel were not informed about the study and therefore not biased. A limitation of this setup was, however, that the time spent in the ambulance was not included in the 24-h. Even though transportation often was short (median transportation time: 37 min, Table 3), patients brought in by EMS were *de facto* observed for fluid administration more than 24 h. It is a limitation that the study did not have valid data on the amount of fluid given in the prehospital setting but only valid data on the totals including prehospital fluids. We are not able to report on neither fluid balance nor number of patients who experienced signs or symptoms of fluid overload.

Also, a large patient population (*n* = 1057) was not included in this study since patients were either discharged within 24 h, died within 24 h, or fluid evaluation was not possible for all 24 h (Figure 1). This, of course, limits the generalizability to all patients with suspected infection. Exclusion of patients discharged within 24 h, may increase especially the investigated population age and comorbidity proportion, since most patients discharged were younger and less sick at admission.

More than half of the patients only had simple infection by definition and were therefore less ill. Less ill patients may be less susceptible to the potential deleterious effects of fluid accumulation and the risk of edema, why fluid administration to these patients may be less controlled and regarded low risk. This may have resulted in especially increased fluid volumes in non-EMS transported patients. On the other hand, even less ill patients often receive fluids in the ambulance anyways if transported by EMS.

This was a *post hoc* analysis, commenced based on observations during the enrollment of patients in the original study (16). No protocol nor statistical analysis plan has been published, and we also did, as mentioned, not calculate a formal sample size *a priori*. However, the original study was based on unpublished data from Aarhus University Hospital: we anticipated that approximately 1,600 patients with suspected infection would present at the three sites within the study period. This anticipated sample size was deemed adequate for the descriptive goal of the current study, and the actual number of patients with suspected infection turned out to be 1,924 patients.

Our findings were overall not surprising but highlights the different pathway and trajectory of interventions that EMS transportation can lead to including higher fluid volumes. The results may, however, partly represent that patients brought in by ambulance are simply a very different patient population, although we tried to account for this by adjusting our analyses. Still, increased fluid volumes were found administered to patients transported by EMS, which could lead to an increased risk of fluid accumulation and edema.

In conclusion, this *post hoc* analysis suggests that patients with suspected infection brought in by EMS received a little more fluid during the first 6 and 24 h of their stay than non-EMS patients after adjusting for age, site, and illness severity. Patients brought in by EMS received more intravenous fluids and less oral fluids than patients arriving on their own. These findings are hypothesis-generating and warrant further investigation. The optimal fluid strategy in the prehospital setting in collaboration with the ED setting in patients with infections is still to be investigated.

## Data availability statement

The raw data supporting the conclusions of this article will be made available by the authors, without undue reservation, on request.

## Ethics statement

The studies involving human participants were reviewed and approved by Danish Patient Safety Authority (case nos. 31-1521-188 and 1-45-70-69-20). Written informed consent for participation was not required for this study in accordance with the national legislation and the institutional requirements.

## Author contributions

MKJ, JK-P, and HK conceived the study idea. MEJ and MKJ collected and analyzed data and wrote the draft for the manuscript. All authors revised the manuscript and approved its submission.
